# Rv1985c, a promising novel antigen for diagnosis of tuberculosis infection from BCG-vaccinated controls

**DOI:** 10.1186/1471-2334-10-273

**Published:** 2010-09-17

**Authors:** Jiazhen Chen, Sen Wang, Ying Zhang, Xiaodi Su, Jing Wu, Lingyun Shao, Feifei Wang, Shu Zhang, Xinhua Weng, Honghai Wang, Wenhong Zhang

**Affiliations:** 1 Department of Infectious Diseases, Huashan Hospital, Fudan University, Shanghai, 200040, China; 2 State Key Laboratory of Genetic Engineering, Institute of Genetics, Fudan University, Shanghai, 200433, China; 3 Department of Molecular Microbiology and Immunology, Bloomberg School of Public Health, Johns Hopkins University, Baltimore, MD, 21205, USA; 4 Institutes of Biomedical Sciences, Fudan University, Shanghai, 200040, China

## Abstract

**Background:**

Antigens encoded in the region of difference (RD) of *Mycobacterium tuberculosis *constitute a potential source of specific antigens for immunodiagnosis. In the present study, recombinant protein Rv1985c from RD2 was cloned, expressed, purified, immunologically characterized and investigated for its potentially diagnostic value for tuberculosis (TB) infection among BCG-vaccinated individuals.

**Methods:**

T-cell response to Rv1985c was evaluated by IFN-γ ELISPOT in 56 TB patients, 20 latent TB infection (LTBI) and 30 BCG-vaccinated controls in comparison with the commercial T-SPOT. *TB *kit. Humoral response was evaluated by ELISA in 117 TB patients, 45 LTBI and 67 BCG-vaccinated controls, including all those who had T-cell assay, in comparison with a commercial IgG kit.

**Results:**

Rv1985c was specifically recognized by cellular and humoral responses from both TB and LTBI groups compared with healthy controls. Rv1985c IgG-ELISA achieved 52% and 62% sensitivity respectively, which outperformed the sensitivity of PATHOZYME-MYCO kit (34%) in detecting active TB (P = 0.011), whereas IFN-γ Rv1985c-ELISPOT achieved 71% and 55% sensitivity in detecting active and LTBI, respectively. Addition of Rv1985c increased sensitivities of ESAT-6, CFP-10 and ESAT-6/CFP-10 combination in detecting TB from 82.1% to 89.2% (P = 0.125), 67.9% to 87.5% (P < 0.001) and 85.7% to 92.9% (P = 0.125), respectively.

**Conclusions:**

In conclusion, Rv1985c is a novel antigen which can be used to immunologically diagnose TB infection along with other immunodominant antigens among BCG-vaccinated population.

## Background

One-third of the world population is infected with *Mycobacterium tuberculosis*. In 2006, there were an estimated 9.2 million new cases of tuberculosis (TB) and 14.4 million prevalent cases of TB [1]. Among people infected with TB bacilli, about 5-10% will become sick or infectious at some time during their life [2]. People with HIV and TB infection are much more likely to develop active TB [1].

The current TB diagnosis still relies on clinical examination and radiography, confirmed by sputum smear microscopy and mycobacterial culture, which often leads delays in treatment due to slow growth of the mycobacteria. Tuberculin skin test (TST) cannot be used as a specific diagnostic test due to the presence of cross-reactive antigens with BCG vaccine and other environmental mycobacteria exposure [3,4]. Moreover, accurate differentiation of latent TB infection (LTBI) from BCG-vaccinated individuals by TST is difficult. Thus, these underscore the need for identifying *M. tuberculosis *specific antigens and developing rapid, specific as well as cost-effective diagnostic tests that can differentiate LTBI from BCG-vaccinated individuals.

Antigens encoded in the region of differentiation (RD) of *M. tuberculosis *constitute a potential source of specific antigens for immunodiagnosis [5-10]. RD2, deleted from BCG substrains derived from the original BCG Pasteur strain during year 1926-1931 [11], encodes 11 ORFs and is conserved in all virulent *M. tuberculosis*. Among them, Mpt64 and Cfp21 are immunodominant antigens and have been used as new protective vaccines and specific diagnostic reagents [9,12-15]. In previous study, Rv1989c, Rv1978, Rv1981c were investigated for T cell-based diagnosis of TB [16,17]. Rv1985c, also encoded by RD2, is a putative transcriptional regulatory protein. Except that Rv1985c was used in a transcriptional study [18], little is known about the immunogenic properties of Rv1985c.

The present study was designed to evaluate both humoral and cellular immune responses to Rv1985c, encoded in RD2 region of *M. tuberculosis*, and its ability to detect active as well as LTBI from BCG-vaccinated individuals.

## Methods

### Study population

Blood samples were collected from totally 229 subjects in this study, which were classified into three groups: active TB patients (TB group, n = 117), LTBI (LTBI group, n = 45) and BCG-Denmark vaccinated healthy controls (HC group, n = 67). The present study is approved by the Ethics Committee from Huashan Hospital, Fudan University. All individuals are Chinese; all patients and guidance had given informed consent. The demographic characteristics of the study populations are described in Table 1.

**Table 1:** The demographic characteristics of the total study population:

Groups		TB	LTBI	HC
**Description**		1. clinical symptom of pulmonary TB	1. very close TB contacts recently that live together	1. healthy, BCG-vaccinated controls;
		2. positive acid-fast bacilli in sputum smears or positive mycobacteria culturing or a suggestive chest X-ray	with *TB *patient 2. positive of T SPOT. *TB *test	2. no prior *M.TB *contact or exposure
		3. (for T-cell tests only ) received DOTS therapy less then 1 month	3. no clinical symptom, nor abnormal chest radiograph	

**Humoral response study**	No. of subjects	117	45	67
	
	Mean age (yr)	46.4	45.1	34.9
	
	Range of age (yr)	6-84	16-76	21-68
	
	Male/female	85/32	13/32	32/35

**Cellular response study**	No. of subjects	56	20	30
	
	Mean age (yr)	46.5	43.6	29.6
	
	Range of age (yr)	11-81	23-74	21-58
	
	Male/female	35/21	4/16	13/17

Pulmonary TB patients were recruited from TB hospitals in 3 regions of China from Chongqing, Jinan and Suzhou. The inclusion were made based on the following criteria: 1) clinical signs and symptoms including fever, cough and productive sputum; 2) positive acid-fast bacilli in sputum smears or positive mycobacterial culture or a suggestive chest X-ray. Since anti-tuberculosis treatment could affect the response of IFN-γ to specific antigens [4,19-21], patients who received chemical therapy more than 1 month were excluded from the T-cell assays.

Forty-five LTBI individuals were recruited based on the criteria including: 1) close TB contacts that lived together with TB patient in recent months; 2) positive T-SPOT^®^. *TB *(Oxford Immunotech, UK) test that indicates TB infection; and 3) no clinical signs or symptoms nor abnormal chest X-ray. All the blood samples were collected before LTBI were treated.

The control group consisted of healthy subjects recruited from students and faculties at Fudan University, China, who had received BCG-Denmark vaccination during childhood and have no history of TB contact. Individuals with known prior exposure were excluded and only healthy (no clinical symptoms) and no contact to TB individuals were collected.

### Study design

Totally, 56 TB patients, 20 LTBI and 30 healthy controls had both T-cell assays and antibody tests. Additional 61 TB patients, 25 LTBI and 37 healthy controls only had antibody tests. T-cell assays include Rv1985c IFN-γ ELISPOT assay and a comparative T-SPOT^®^. *TB *assay, which use two other immunodominant antigens, 6-kDa Early Secretory Antigenic Target (ESAT-6) and 10-kDa Culture Filtrate Protein (CFP-10). Antibody tests include Enzyme-Linked ImmunoSorbent Assay (ELISA) to Rv1985c and a comparative commercial PATHOZYME-MYCO IgG kit (Omega Diagnostics, UK), which uses two immunodominant antigens, lipoarabinomannan (LAM) and 38-kDa antigen.

### Cloning, expression and purification of Rv1985c

RNA was extracted from stationary phase *M. tuberculosis *H37Rv cells and cultured in Middlebrook 7H9 medium, as previously described [22]. RT-PCR was performed to confirm the expression of Rv1985c in *M. tuberculosis*, using the ThermoScript RT-PCR system (Invitrogen, CA, USA), with the specific primers Rv1985c-F (5'-ATCCATATGGTGGATCCGCAGCTTGACGGT) and Rv1985c-R (5'-TATGTCGACACCCGGTCGGCGGCG), according to the manufacturer's instructions. NdeI and SalI restriction sites were incorporated at the 5'-end of the forward and reverse primers, respectively. The amplicon containing *rv1985c *was cloned at the NdeI and SalI sites of pET30a vectors with a C-terminal six-histidine tag. The construct was verified the correct insert and orientation by Prism 377 DNA sequencer (Applied Biosystems, Warrington, UK).

After the construct comprising *rv1985c *was transformed into *Escherichia coli Rosetta *(DE3), His-tagged Rv1985c protein was expressed by 1.0 mM Isopropyl-β-D-galactopyranoside (Sigma) and purified under native conditions by His-Bind Column (Novagen) according to manufacturer's recommendation. The purified Rv1985c was dialyzed and stored at -20°C in 25 mM HEPES (pH 7.4) supplemented with 150 mM NaCl, 0.1 mM DMSF and 10% glycerol. The fractions were also analyzed by SDS-PAGE and proteins were quantified by Bradford reagent (Bio-Rad, UK). A HPLC-MS study was applied to determine purity.

### IFN-γ ELISPOT assays

Due to limitation of resources, totally 56 TB patients, 20 LTBI and 30 healthy controls accepted and had T-cell assays simultaneously, which include Rv1985c IFN-γ ELISPOT assay and a comparative T-SPOT^®^. *TB *assay. IFN-γ Rv1985c-ELISPOT assay was performed blinded according to manufacturer's instruction [23]. A totally 2.5 × 10^5 ^peripheral blood mononuclear cells (PBMC) were added in duplicates in micro-wells. Rv1985c was used at a final concentration of 10 μg/ml (optimized). PHA mitogen at 5 μg/ml was employed as a positive control and culture media as negative control. The plate was incubated at 37°C, 5% CO_2 _incubator for 20-24 hr. Positive Rv1985c-ELISPOT test wells were defined as containing greater than or equal to 6 spots and the number was at least twice as many spots as the negative control. For analysis, PHA control wells were required to have at least 50 SFUs (spot-forming units), and negative control wells were required to have fewer than 10 SFUs.

The T-SPOT.*TB *assay enumerating effector T cells responding to stimulation with ESAT-6-and CFP-10 was also performed as a comparative ELISPOT method according to the manufacturer's instructions [4]. The spot was counted by technicians blinded to the subject identifiers using an automated ELISPOT Reader (AID systems, Germany). According to the manufacturer's instructions, either ESAT-6-or CFP-10-stimulated wells containing greater than or equal to 6 spots with the number at least twice as many spots as the negative control were considered positive.

### Serological tests

Totally, 117 TB patients, 45 LTBI and 67 healthy controls including all those who had T-cell assay accepted and had antibody tests simultaneously which include Rv1985c-ELISA and PATHOZYME-MYCO IgG kit (Omega Diagnostics, UK). IgG-and IgM-ELISA was performed to determine the humoral immune response to recombinant Rv1985c by 96-well microtitre plates as previously described [24]. Microtitre plates were coated with 0.5 μg/100 μl of Rv1985c. The sera were diluted 500-fold for IgG-ELISA and 100-fold for IgM-ELISA, distributed in microtitre plates and incubated for 1 h at 37°C. Each serum was repeated three times. The IgG-and IgM-ELISA results were analyzed using cut-off values equal to the mean OD for the healthy control serum samples plus two standard deviations. Any sample exhibiting absorbance above the cut-off value was considered positive.

The PATHOZYME-MYCO IgG kit measuring the level of IgG antibody to two antigens, LAM and 38-kDa antigen was also performed as a comparison. The assay was done according to manufacturer's recommendation as described previously [25,26]. Four standards (with 2, 4, 8 and 16 sero-units/ml) were provided for generation of a semi-logarithmic reference curve. The units result was interpreted by extrapolating the optical density (OD) from the curve and multiplying by 100 (diluted 1/100). According to the manufacturer's instructions, sera of higher than 400 units/ml were considered IgG-positive.

### Statistical analysis

For statistical analysis, the OD differences among groups were calculated by one-way ANOVA Newman-Keuls test. The SFU difference of among groups was analyzed by Kruskale-Wallis one-way analysis Dunn's post-test. The sensitivities of different tests were analyzed by McNemar's chi-square test. In all analysis, P values ≤ 0.05 were considered statistically significant.

## Results

### Expression and purification of *M. tuberculosis *Rv1985c protein

The expression of Rv1985c at the mRNA level was verified by RT-PCR study. An expected 930-bp PCR product was revealed by 1.0% agarose gel electrophoresis, and no PCR product was detected in the absence of reverse transcriptase. These data indicated that Rv1985c was expressed during the growth in vitro (data not shown).

**Figure 1:**
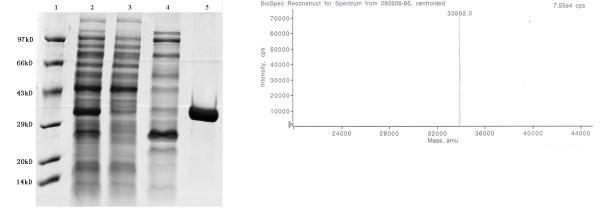
**Purification and analysis of recombinant Rv1985c**. (A) Electrophoresis analysis on a 12% SDS-PAGE gel stained with Coomassie blue; Lane 1, Takara low molecular weight marker; Lane 2, crude ***E. coli ***supernatant fraction before application to column (induced with IPTG); Lane 3, flow-through fraction of His-bind resin; Lane 4, fraction washed with 120 mM imidazole; Lane 5, fraction eluted with 250 mM imidazole. Molecular weight of Rv1985c is approximately 33.8 kDa and protein marker sizes are indicated on the side of gel. (B) Mass-fingerprinting protein assay of 33.9-kDa by MALDITOF.

The recombinant Rv1985c containing a minimal C-terminal 6-Histidine tag verified by probing with monoclonal anti-His tag antibody (data not shown) was largely present in the supernatant fraction. The purified protein yielded 4.1 mg/liter of culture and a single 34-kDa protein was observed in SDS-PAGE gel (Figure 1A). HPLC analysis confirmed the purity of the protein to 98.7%. The approximate molecular weight for recombinant Rv1985c was 33.892 kDa (Figure 1B), which is in accordance with the theoretical mass prediction of 33.886 kDa.

### Specific T-cell response to Rv1985c in TB and LTBI groups compared with healthy controls

**Figure 2:**
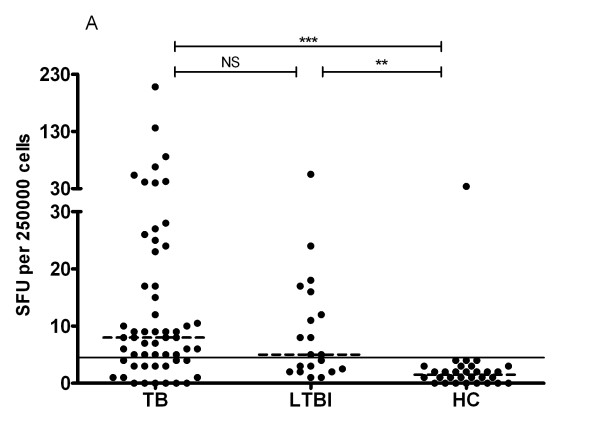
**ELISPOT showed that PBMC from both TB and LTBI groups developed stronger IFN-γ producing response to Rv1985c antigens than those from HC (healthy controls) group**. PBMC from TB, LTBI, and HC individuals were stimulated overnight with recombinant Rv1985c and IFN-γ producing T cells were detected by ELISPOT. Results of individual response are expressed as spot forming units (SFU) per 2.5 × 10**^5 ^**PBMCs. Horizontal lines indicate the median of SFU of each group. SFU greater than or equal to 6 was considered positive (indicated by the dotted line). P-values of SFU difference between either two groups were shown above the plots determined by Kruskale-Wallis one-way analysis Dunn's post-test. ***, P < 0.001; **, P < 0.01; *, P < 0.05; NS: P-value is not significant.

**Figure 3:**
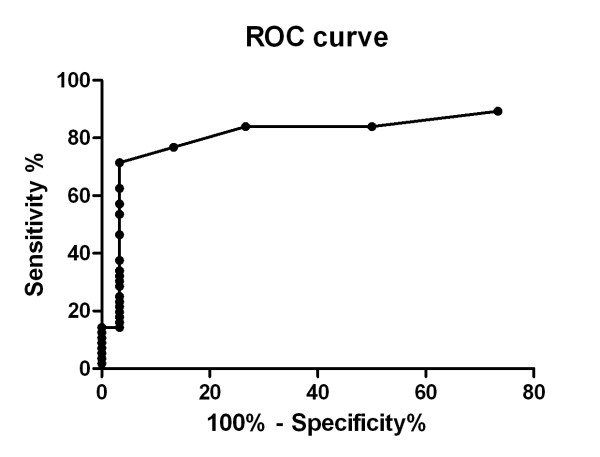
**Receiver-operator characteristics (ROC) curve for Rv1985c for diagnosis of active TB by T-cell response**. The curve describes the association between sensitivity and specificity at different thresholds of the study.

The SFU of TB and LTBI groups were significantly higher than that of HC group (P < 0.001) (Figure 2), indicating PBMC from both TB and LTBI groups developed stronger T-cell responses than those of control group. However, no statistic difference in SFU was observed between TB and LTBI groups (P = 0.25). Figure 3 showed the ROC curve of Rv1985c, which described the relationship between the sensitivity and specificity at any cut-off level. The area of the curve was 0.836 (95% CI, 0.746-0.927). The cut-off level was set at 4.5, giving the optimal combination of sensitivity and specificity. Using the cut-off level, 40/56 (71%) TB patients, 11/20 LTBI (55%), and 1/30 controls (3%) were positive by the T-cell assay.

### Comparison of Rv1985c-ELISPOT test with T-SPOT. TB Test

T-SPOT.TB test was performed comparatively in the same category groups. Totally 46 patients (82.1%) had positive responses to ESAT-6 and 38 (67.9%) had positive responses to CFP-10 (Table 2). In contrast, none of healthy controls had positive result. In this study, T-SPOT. TB achieved 85.7% sensitivity (either positive to ESAT-6 or CFP-10).

**Table 2:** Characterization of IFN-γ ELISPOT in response to recombinant Rv1985c and the peptides mixture from ESAT-6 and CFP-10 in TB patients, LTBI and Healthy controls groups:

	TB (n = 56)				LTBI (n = 20)				HC (n = 30)			
**Antigens**	**Mean SFU** **± standard error**	**No. positive^1^**	**Sensitivity^2 ^(%)**	**95% CI (%)**	**Mean SFU** **± standard error**	**No. positive^1^**	**Sensitivity^3 ^(%)**	**95% CI (%)**	**Mean SFU** **± standard error**	**No. positive^1^**	**Specificity^4 ^(%)**	**95% CI (%)**

Rv1985c	19.3 ± 4.7	40	71.4	57.8 to 82.7	10.0 ± 2.8	11	55	31.1 to 78.9	2.6 ± 1.1	1	96.7	82.8 to 99.9

ESAT-6	66.59 ± 9.3^5^	46	82.1^6^	70.6 to 91.2	37.6 ± 12.0	15	75	54.2 to 95.8	0.77 ± 0.20	0	100.0	88.4 to 100.0

CFP-10	132.8 ± 22.2^5^	38	67.9^7^	54.9 to 80.2	77.9 ± 24.0	15	75	54.2 to 95.8	1.0 ± 0.25	0	100.0	88.4 to 100.0

^1^: Positive was defined as SFU greater than or equal to 6 in 2.5 × 10^5 ^cells for ESAT-6 and CFP-10, and greater than 4.5 in 2.5 × 10^5 ^cells for Rv1985c;

^2^: Percentage of responding patients out of all TB patients tested;

^3^: Percentage of responding individuals out of all LTBI tested;

^4^: Percentage of true-negative healthy controls out of all healthy control individuals tested;

^5^: Statistically higher SFU than SFU of Rv1985c by two-tailed paired Mann-Whitney test, p < 0.001;

^6^: Statistical significance was determined in respect to sensitivity of Rv1985c by McNemar chi-square test, p = 0.02;

^7^: Not significant in respect to sensitivity of Rv1985c by McNemar chi-square test, p = 0.52.

Compared with the sensitivity of Rv1985c (71%) in detecting active TB, ESAT-6 and CFP-10 had the equivalent sensitivity level (P = 0.18 and 0.82 respectively). Both ESAT-6 and CFP-10 showed similarly high specificity (both 100%) compared with Rv1985c (96.7%) without statistically significant difference. However, the strength of T-cell response to Rv1985c was lower than those of ESAT-6 and CFP-10 (both P < 0.05).

To maximize the sensitivity of a future diagnostic reagent, we investigated to combine Rv1985c with ESAT-6 and CFP-10 in TB detection. The results showed addition of Rv1985c increased the sensitivity of single antigen ESAT-6, CFP-10 and combined antigens of ESAT-6/CFP-10 in detecting TB from 82.1% to 89.2% (P = 0.125), 67.9% to 87.5% (P < 0.001) and 85.7% to 92.9% (P = 0.125), respectively.

### Serological response to Rv1985c in TB and LTBI groups compared with healthy controls

**Figure 4:**
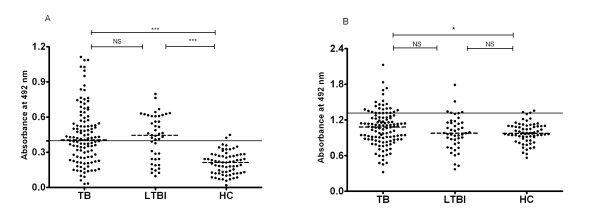
**Comparison of antibody IgG (4-A) and IgM (4-B) responses to recombinant protein Rv1985c among different categories of TB patients, LTBI and HC**. Dotted lines on each group indicate the median value. Horizontal lines on each graph indicate the cut-off value, which was determined by the mean absorbance plus 2 SD by use of healthy control sera. P-values of absorbance difference between either two groups were shown above the plots determined by one-way ANOVA Newman-Keuls test. ***, P < 0.001; **, P < 0.01; *, P < 0.05; NS: P-value is not significant.

Figure 4 showed the IgG (4A) and IgM (4B) antibody OD values to Rv1985c obtained from 117 TB patients, 45 LTBI and 67 healthy controls. The data demonstrated that the sera from TB and LTBI groups had higher IgG antibody responses than those from HC group (p <0.0001) (Figure 4A). However, only TB group (P = 0.040) but not LTBI group (P = 0.97) had significantly higher IgM response than that in healthy donors (Figure 4B).

Totally, 61 of 117 TB patients, 28 of 45 LTBI and 2 of 67 HC had greater IgG values than the cut-off values, whereas only 23 of 117 TB patients, 5 of 45 LTBI and 2 of 67 HC had greater IgM value than the cut-off values (Figure 4). The sensitivity of Rv1985c-ELISA IgG in detecting TB and LTBI were 52.1% and 62.2% respectively, and the sensitivities of Rv1985c-ELISA IgM in detecting TB and LTBI were 19.7% and 11.1%, respectively (Table 3). The specificity of Rv1985c-IgG and Rv1985c-IgM response in healthy controls was both 97.0%. IgG antibody responses to Rv1985c were observed stronger and more specific than IgM antibody responses (Figure 4), and serum sample of IgG test were diluted 1:500 (optimized) instead of 1:100.

**Table 3:** Seroreactivity of recombinant proteins in sera from TB patients, LTBI and HC

	TB (n = 117)		LTBI (n = 45)		HC (n = 67)	
**Antigens**	**No. Positive^1^**	**Sensitivity^2 ^(%)**	**No. Positive^1^**	**Sensitivity^3 ^(%)**	**No. Positive^1^**	**Specificity^4 ^(%)**

PATHOZYME-MYCO IgG	40	34.2	NT	NT	1	98.5

**Rv1985c **IgG	61	**52.1**^5^	28	62.2%	2	97.0

Rv1985c IgM	23	19.7	5	11.1%	2	97.0

**Rv1985c **IgG + IgM	73	**62.4**^5^	29	64.4%	3	95.5

**Rv1985c**+ PATHOZYME-MYCO IgG	82	**70.1**^5^	NT	NT	3	95.5

^1^: For 1985c positive sera, defined as cases for which the optical density (OD) generated by the serum was greater than the mean OD of the healthy control sera plus two standard deviations. For PATHOZYME-MYCO IgG positive sera, defined as cases of more than 400 units per ml, which was calculated by extrapolating the OD from the standard curve and multiplying by 100;

^2^: Percentage of responding patients out of all TB patients tested;

^3^: Percentage of responding patients out of all LTBI tested;

^4^: Percentage of true-negative healthy controls out of all healthy control individuals tested;

^5^: Statistical significance was determined in respect to sensitivity of PATHOZYME-MYCO IgG by McNemar chi-square test, p < 0.01;

NT: not tested.

### Rv1985c-IgG ELISA in Comparison with PATHOZYME-MYCO IgG kit

To better evaluate the humoral antigenic feature of Rv1985c, the IgG response was compared with the commercial PATHOZYME-MYCO ELISA IgG kit for diagnosis of active TB. The sensitivity and specificity of PATHOZYME-MYCO IgG kit in the detection of active TB were 34.2% and 98.5%, respectively. Comparing with Rv1985c-IgG ELISA, PATHOZYME-MYCO IgG test had lower sensitivity (34.2%) than Rv1985c-IgG ELISA (52.1%) in detecting TB with significant difference by McNemar chi-square test (P = 0.011) (Table 3).

## Discussion

In the present study, cloning, expression, purification and both humoral and cellular immune responses to recombinant antigen Rv1985c of *M. tuberculosis *were evaluated for the first time in active TB patients, LTBI individuals and BCG-vaccinated healthy controls. Rv1985c was specifically recognized by both cellular and humoral responses from the TB and LTBI groups compared with the healthy controls. IFN-γ Rv1985c-ELISPOT achieved 71% and 55% sensitivity in detecting active and latent TB, respectively, and 96.7% specificity in healthy controls in this test, whereas Rv1985c-ELISA IgG achieved 52% and 62% sensitivity in detecting active and latent TB, respectively, and 97.0% specificity in the healthy controls. Since one-third of population is infected with TB in the world, especially in high epidemic region, it would therefore be meaningful if a cost-effective and sensitive sero-diagnostic test capable of identifying LTBI from healthy controls could be developed. To some extent, data showing that Rv1985c-IgG ELISA achieved a sensitivity of 62% in detecting LTBI and could distinguish LTBI from healthy BCG-vaccinated individuals is promising.

In the study, latently infected individual was determined by measurement of T-SPOT *TB *instead of TST in high risk BCG-vaccinated population. Specific cellular response to Rv1985c can be found not only in individuals with active TB disease, but also in individuals with latent *M. tuberculosis *infection. Recently, antigen TB 7.7 and Rv3879c were determined to increase diagnostic sensitivity in Quantiferon TB Gold In-tube and T-SPOT. TB assays when used in combination with ESAT6 and CFP10 [27,28]. Since the T-cell response to Rv1985c had equivalent sensitivity to CFP-10 and ESAT-6, it is very possible to improve the diagnostic performance of ESAT-6 and/or CFP-10 by addition of Rv1985c. Though the additive effect made for ESAT-6 alone or ESAT-6 with CFP-10 was not statistically significant among 56 TB patients, the combination of Rv1985c with CFP-10 could improve sensitivity of CFP-10 with statistical significance and the specificity remained equivalently high.

In addition, to evaluate the antigenic feature of Rv1985c, both T-cell response and B-cell response to Rv1985c were compared with other antigens. Compared Rv1985c IgG-antibody response with PATHOZYME-MYCO IgG kit, Rv1985c showed higher sensitivity than LAM and 38-kDa antigens in diagnosing active TB, suggesting that Rv1985c may be a better serodiagnostic reagent than LAM and 38-kDa in terms of distinguishing TB patients from healthy BCG-vaccinated individuals. Although a sensitivity of 52% is not high enough as a stand-alone serodiagnostic test for TB, it could be used with other immunodominant antigens in combination. When Rv1985c-IgG tests were combined with Rv1985c-IgM tests, the sensitivity was improved from 52.1% to 61.4% (P < 0.001) with equivalent high specificity (95.5%) (Table 3). The combination of Rv1985c and PATHOZYME-MYCO IgG yielded 70% sensitivity, which is similar to TST (77%) [29]. Since the specificity of the combination (96%) was superior to TST (59%) in BCG-vaccinated population [29], this system could be used to replace TST. It is also noted that the sensitivity of PATHOZYME-MYCO IgG (34%) was slightly lower than the other studies, ranged from 41% to 49% [26,30,31]. Since the serological response to a specific antigen often depends on the geographical location and ethnic background of the population being studied [32], the observed small variation in performance of PATHOZYME-MYCO IgG is understandable.

It is known that T-cell response to most immunodominant antigens of *M. tuberculosis*, like ESAT-6 and CFP-10, could not differentiate active TB infection with latent infection [29]. However, whether humoral response to immunodominant antigens can differentiate active TB infection with LTBI is less known [33]. On the basis of our data showing that no statistical difference was observed between TB and LTBI groups in humoral response against Rv1985c, we suggest that the humoral response to some immunodominant antigens could either not be able to differentiate active TB with LTBI. Consistently, a recent study showed that ESAT-6 and CFP-10 antibodies are present not only in active TB patients but also in latent infection individuals, particularly in areas with high levels of exposure to *M. tuberculosis *[33]. These studies may underlie the insufficient specificity of the sero-diagnostic tests designed to detect active TB and used in TB epidemic regions [30,34,35].

Surprisingly, the humoral response to Rv1985c did not correlate at all with the cellular response (P = 0.87) in the LTBI group. A possible explanation is that person-to-person variation of antigen recognition, rather than recognition of particular antigens, is the key attribute of humoral immunity in TB [36].

Since RD2 is absent in BCG strains acquired from the Pasteur Institute after 1931, including vaccine BCG-Phipps, BCG-Tice, BCG-Frappier, BCG-Prague, BCG-Connaught, BCG-Glaxo, BCG-Pasteur and BCG-Denmark, which is now widely used in China, Rv1985c is not expected to be cross-reactive [11]. However, we observed 1/30 healthy subjects had positive Rv1985c-ELISPOT response and 2/67 had positive IgG-antibody response to Rv1985c. Rv1985c is a putative lysR protein encoded by RD2 region of the *M. tuberculosis *genome. Rv1985c may be present in a few BCG, such as BCG-Russia, BCG-Moreau, BCG-Japan, BCG-Sweden and some NTM strains [11], which may cause some immune response to the antigen and underlie the 3% positive response in healthy controls in both tests. It should also be noted that the T-cell response of Rv1985c was weaker than those of RD1-encoded antigens. Some individuals were just above the cut-off value. According to the recent T-SPOT. *TB *guild of diagnosing TB infection, when spot count is 5, 6 or 7, the results should be considered borderline or equivocal. If we use this criteria in Rv1985c, 10 of 56 (18%) TB and 2 of 21 (10%) LTBI individuals of Rv1985c-ELISPOT assay should be interpreted as borderline, which reflects relative weakness of the T-cell response of the antigen.

Furthermore, because of the difficulty in recruit, three groups were recruited from different areas. TB and LTBI were recruited from Chongqing, Jinan and Suzhou, whereas the HC were from Shanghai. Although same in ethnics, different in age and areas could result in difference in background immune responses, which could affect the cut-off levels and consequently the sensitivity and specificity of the assays.

Finally, since LTBI was partly defined by a positive T-SPOT.TB, to compare Rv1985c-ELISPOT with T-SPOT.TB to evaluate the antigen in diagnosing LTBI has some drawbacks. Thus, due to lack of a reference standard, we didn't compare the two assays in diagnosing LTBI. The value of the antigen should be better recognized and evaluated by prognostic study, such as predicting subsequent development of active TB disease [37,38]. Because this is the first study to evaluate Rv1985c, further validation on diagnostic criteria is still required.

## Conclusions

In conclusion, Rv1985c it a novel antigen which is specifically recognized by cellular and humoral responses from both TB and LTBI individuals compared with healthy individuals. Reactivity towards Rv1985c can be used to immunologically diagnose TB infection individuals along with other immunodominant antigens among BCG-vaccinated population.

## Competing interests

The authors declare that they have no competing interests.

## Authors' contributions

JC carried out the protein study, immunology study, data collection, data analysis and drafted the manuscript. SW participated in the protein purification, ELISA study and data collection. YZ participated in the study design and helped the manuscript. XS participated in the protein purification, data collection and analysis. LS participated in the T-SPOT TB assay. JW participated in the immunology study. SZ participated in the organization of TB patients and T-SPOT TB assay. FW participated in the organization of healthy controls. XW contributed to the organization of TB patients. HW contributed to the study design. WZ was responsible for the study design, organization and revised the manuscript. All authors read and approved the final manuscript.

## Pre-publication history

The pre-publication history for this paper can be accessed here:

http://www.biomedcentral.com/1471-2334/10/273/prepub
